# Revealing the Mechanism of Friedelin in the Treatment of Ulcerative Colitis Based on Network Pharmacology and Experimental Verification

**DOI:** 10.1155/2021/4451779

**Published:** 2021-11-02

**Authors:** Bei Shi, Suxian Liu, Aoshuang Huang, Mengyun Zhou, Boyun Sun, Hui Cao, Jingyi Shan, Bo Sun, Jiang Lin

**Affiliations:** ^1^Department of Gastroenterology, Longhua Hospital Shanghai University of Traditional Chinese Medicine, Shanghai, China; ^2^Institute of Digestive Diseases, Longhua Hospital, Shanghai University of Traditional Chinese Medicine, Shanghai 200032, China; ^3^Department of Pathology, Longhua Hospital Shanghai University of Traditional Chinese Medicine, Shanghai, China

## Abstract

**Objectives:**

Ulcerative colitis (UC) is a chronic inflammatory disease affecting the colon, and its incidence is rising worldwide. This study was designed to uncover the healing effect of friedelin, a bioactive compound against UC through bioinformatics of network pharmacology and experimental verification of UC model mice.

**Materials and Methods:**

Targets of friedelin and potential mechanism of friedelin on UC were predicted through target searching, PPI network establishing, and enrichment analyzing. We explored effects of friedelin on dextran sulfate sodium (DSS)-induced colitis. Severity of UC was investigated by body weight, disease activity index (DAI), and length of the colon. Inflammation severity was examined by determination of proinflammatory and anti-inflammatory cytokines. The numbers of autophagosome around the epithelial cells were observed by autophagy inhibition via a transmission electron microscope. The expressions of autophagy-related ATG5 protein and AMPK-mTOR signaling pathway were determined by immunofluorescence staining.

**Results:**

In this study, 17 potential targets of friedelin and 1111 UC-related targets were identified. 10 therapeutic targets of friedelin against UC were acquired from overlapped targets of UC and friedelin. PPI network construction filtered 14 core targets through target amplification and confidence enhancement. The results of molecular docking showed that the docking scores of the top 5 active targets were higher than the threshold values. Gene ontology (GO) and Kyoto Encyclopedia of Genes and Genomes (KEGG) pathway analyses were carried out, showing friedelin alleviates UC through anti-inflammatory pathways and molecular function of autophagy. Subsequently, animal-based experiments revealed the intraperitoneal injection of friedelin ameliorated DSS-induced body weight loss, DAI decrease, colon length shortening and colonic pathological damage with lower myeloperoxidase and proinflammatory cytokines (IL-1*β* and IL-6) and higher IL-10 levels, and more autophagosomes in transmission electron microscope results. The AMPK-mTOR signaling pathway plays important role in the friedelin's effect in autophagy as KEGG pathway result and experiment verification. Furthermore, the 3 ma validated the role of autophagy as an improvement in the friedelin's pharmacologic effect to UC model mice.

**Conclusions:**

Friedelin ameliorated DSS-induced colitis in mice through of inflammatory inhibition and regulation of autophagy.

## 1. Introduction

Ulcerative colitis (UC) and Crohn's disease (CD), which both belong to chronic inflammation of the intestine, are the two main subtypes of inflammatory bowel disease (IBD) in clinical work [1]. The epidemiology of IBD is changing throughout the world at the turn of the 21st century. The incidence is stabilizing in western countries; however, incidence rates in newly industrialized countries have been increasing quickly, attributing to rapid socioeconomic development [2]. The main feature of UC is a recurrence cycle with mucosal ulceration and diffuse colonic inflammation [3]. Meanwhile, long-term UC involving at least a third of the colon is associated with an increased risk of colorectal cancer [3] and becoming a public health burden globally.

As we all know, the inflammatory response plays important roles in the UC pathogenesis [4]. Several immune cells and immune-regulatory proteins participate in the disturbance of intestine immune system, which accounts for activation and augmentation of inflammation cascade in UC. Commonly, the intestinal epithelial cells are the first barrier of any intestinal disease. Once the epithelial cells are damaged by external injury, such as excessive stress, mechanical damage, and chemical stimulation, the intestinal pathogens may enter the intestinal tract, trigger antigen presenting cells, and transform the immature T cells into differentiated effector T cells, for example, Th1, Th2, Th17, and natural killer T cells [5]. These cells released a variety of proinflammatory cytokines which caused the injury of the colon.

According to its mechanism, anti-inflammation and immunosuppressive agents are the main strategies for UC treatment, which comprise mesalazine (5-amino salicylic acid, 5-ASA) and corticosteroids [6]. In addition, biological agents and small-molecule Janus kinase inhibitors are also a therapeutic choice of this disease [7]. Surgery is the last option, but no matter drugs or surgery commonly leads to the short or long complications. The new pharmacology approaches are needed for adjunct treatment. Nowadays, growing studies reveal that autophagy is tightly related to UC [7]. The cell death and regeneration are bound up with mucosal ulceration cycle [8]. Autophagy is a type 2 cell death in the cell. Therefore, the autophagy becomes a novel therapy target for UC, which attracts interests of the scientists for new drugs development.

The friedelin (friedelan-3-one) is a pentacyclic triterpene isolated from multiple plants. It can be found in the *Aristotelia chilensis* leaves (Elaeocarpaceae) [9], *Cannabis* roots [10], and *Maytenus ilicifolia* leaves [11], which were used in folk medicine to treat pain and inflammation. The friedelin is particularly rich in cork tissues from trees [12]. The friedelin exhibited a variety of biological activities, for example, cytotoxic in human MCF-7 breast cancer cell [13], anti-inflammatory, analgesic, and antipyretic [14], low antimicrobial [15], human liver cytochrome P450 inhibition [16], vasodilator effect [11], and antioxidant effect [17]. In particular, the friedelin was reported with gastroprotective activity [18, 19]. The friedelin exerted antigastric ulcer ability in rats [20]. More importantly, this report also revealed that friedelin is a relative safe agent up to 80 mg/kg in acute toxic test in rats [20], while multitarget mechanism research to analyze the treatment of friedelin against UC is still needed.

Network pharmacology is an emerging field of pharmacology, which utilizes network analysis of drug action as one of its approaches, and network pharmacology is a method by integrating systems approaches, computational and experimental methods to illuminate the molecular mechanisms of drug [21, 22]. Because network pharmacology can provide a good understanding of the principles of network theory and systems biology, it has been considered to be the next paradigm in drug discovery [23].

This study aimed to explore the curative effect of friedelin against UC and to execute it through network pharmacology and experimental verification (Supplementary Figure S1). The friedelin-related targets and UC-related targets were screened from a range of pharmacology platforms, disease databases, and published articles. PPI network was constructed to amplify the targets and enhance confidence. Further, GO functional and KEGG enrichment analyses were performed to elucidate the mechanisms of friedelin in the treatment of UC. Experiments revealed that friedelin attenuated inflammatory responses and improve autophagy amount in DSS-induced mice. Besides, the curative effects were counteracted once 3 ma, an autophagy inhibitor, was added. This study provides innovative ideas for the clinical treatment of UC, as well as a new strategy for developing novel therapeutic drugs.

## 2. Materials and Methods

### 2.1. Network Pharmacology

The targets of friedelin were gathered in Swiss Target Prediction System [24] (http://www.swisstargetprediction.ch/). We overlapped the identified targets of UC from Gene Cards database [25] (https://www.genecards.org/) and DrugBank database [26] (https://go.drugbank.com/); the targets were searched by making “ulcerative colitis” as key word. The potential targets of friedelin against UC were integrated with targets of friedelin and UC.

Protein-protein interaction (PPI) data was acquired from the STRING database (http://string-db.org) [27], with the species limited to “*Homo sapiens*,” 20 interactors to show in the 1st shell, and the cutoff confidence score set at >0.9 to obtain the highest confidence data. PPI networks were established and visualized using Cytoscape visualization software (http://cytoscape. org/, ver. 3.8.2) [28]. PPI networks for friedelin and UC targets were constructed, and an intersection was performed to identify the targets of friedelin against UC. Using TCMNPAS system [29] and AutoDock [30] software, the docking energy and the docking locus between the overlapped proteins and friedelin were presented. Enrichment analyses were subsequently performed using Metascape [31] (https://metascape.org/gp/index.html).

### 2.2. Reagents and Chemicals

The dextran sulfate sodium (DSS) was obtained from MP Biomedicals (Santa Ana, CA, USA). Mesalazine was produced by Sunflower Pharmaceutical group, Jiamusi Lu Ling Pharmaceutical Co., Ltd. (Liaoning, China). Friedelin was brought from NATURE STANDARD (Shanghai, China). Autophagy inhibition was brought from Selleck (Shanghai, China). Fecal occult blood test kit was obtained from Beijing Huashengyuan Medical Science and Technology Co., Ltd. (Beijing, China). The myeloperoxidase (MPO) determination kit was gained from JianCheng Bioengineering Institute (Nanjing, Jiangsu, China). Primary antibodies for ATG5, AMPK, and mTOR were purchased from CST (Cell Signaling Technology Corporation) and Abcam. The ELISA kits of proinflammatory cytokines, hematoxylin-eosin (HE), and periodic acid-Schiff (PAS) reagents were provided by Beyotime (Nantong, Jiangsu, China). Other unmentioned reagents were of analytical grade.

### 2.3. Animal

C57BL/6 mice (All males) at 20–22 g were provided by Shanghai Slac Laboratory Animal Co., Ltd. and housed in the Laboratory Animal Center of Shanghai University of Traditional Chinese Medicine at temperatures of 20–25°C, humidity of 40 ± 5%, and 12 h light/12 h dark cycle environment at SPF grade. The approval for this animal study was provided from Laboratory Animal Ethics Committee of Shanghai University of Traditional Chinese Medicine (Grant Number: PZSHUTCM18121406). The animal study was conducted according to the Declaration of Helsinki and the Use of Laboratory guidelines issued by the Chinese Council on Animal Care.

### 2.4. Group Design, Model Establishment, and Friedelin Treatment

After 3 days of accommodation, mice were randomly assigned into 7 groups (*n* = 6): (1) normal control group (NC), (2) DSS model group (MD), (3) low-dose friedelin group (14 mg/kg/d), (4) medium-dose friedelin group (28 mg/kg/d), (5) high-dose friedelin group (42 mg/kg/d), (6) positive drug group treated with mesalazine (100 mg/kg/d, PD) following previous literature [32], and (7) high-dose friedelin group (42 mg/kg/d) combined with the 3 ma (a common autophagy inhibitor, 10 mg/kg/d) [33]. All groups were fed standard diet. Eight days before friedelin administration, the control group received normal sterilized drinking water. The other 6 groups received 5% DSS involved water to induce UC. Meanwhile, among the friedelin groups, the friedelin was given to mice as desired dose using intraperitoneal injection (i.p.) once each day. The high-dose friedelin (42 mg/kg/d) combined with the 3 ma group was injected with 3 ma at 10 mg/kg/d.

### 2.5. Evaluation of Clinical Parameters of Colitis

The weight loss, feces property, and symptom of hematochezia in mice were recorded per two days. Meanwhile, the general condition of mice under this study, including activity, eating, and movement, was observed. DAI was evaluated following the reported method based on some aspects: body weights changes, stool hemoccult, and consistency [34]. Twenty-four hours after last friedelin administration, mice were sacrificed using cervical dislocation after excessive anesthesia.

### 2.6. Direct and Histological Observation

After execution of mice, the murine colon was harvested from the ileocecal junction to the anal verge. The murine colon was directly observed to explore potential necrosis and bleeding. The length of colon was captured using a camera finally. After record, the murine colon tissues were fixed, embedded in paraffin, and cut into 5 *μ*m sections. Then, the colon tissues were stained by hematoxylin and eosin (HE) and observed using a microscope (MF31, Mingmei, Guangzhou) [35]. The histological score was evaluated by a third-party pathologist who did not participate in the experiment based on the inflammation, lesion depth, and extent in the colon tissues. In addition, the PAS staining was performed to observe the histological changes of colon tissues, especially the goblet cell numbers [36].

### 2.7. Biochemical Assays

ELISA was employed to assess the cytokines and MPO in colon. The colon tissues of mice were harvested and weighted. The cytokines levels of interleukin-6 (IL-6), interleukin-1*β* (IL-1*β*), and interleukin-10 (IL-10) were quantified by ELISA kits following manufacture instruction separately. Meanwhile, the MPO was evaluated as this formula: MPO performance (U/g) = sample OD values − normal control OD values)/11.3 × sample weight (g) [37].

### 2.8. Immunofluorescence (IF) Staining

The immunofluorescence staining was employed to investigate the intrinsic mechanism of friedelin treatment [38]. Concisely, the colon segments were collected quickly, embedded in OCT, and frozen upon the liquid nitrogen. Then, the colon tissues were cut into 4 *μ*m section at −20°C. After blockage with Quick Block TM Blocking Buffer, tissues were incubated with p-AMPK anti-mice antibody (1 : 50, Cat#ab23875, Abcam), p-mTOR anti-mice antibody (1 : 50, Cat#5536, CST), or ATG5 anti-mice antibody (1 : 50, Cat#ab108327, Abcam) overnight at 4°C. After washing using PBS, tissues were reincubated at 37°C for 20 min with donkey IgG Alexa Flour 488 secondary antibody (1 : 100, Cat#A-11029, Thermo Scientific). The slides were stained with DAPI before observation. The fluorescent images were captured using microscope (3DHistech Ltd., Budapest, Hungary). The immunointensity of green was analyzed.

### 2.9. Transmission Electron Microscopy (TEM)

TEM was employed to explore the activity of autophagosome in the colon cells [39]. The colon tissues were fixed with 2.5% glutaraldehyde and consequently 0.1 M PBS washed 3 times. The tissues were postfixed with 1% osmium tetroxide for 2 h, processed by a graded dehydrated using ethanol at 30%, 50%, 70%, 90%, 95%, and 100%, permeated, and embedded. Then, the colon tissues were cut into ultrathin sections at 50 nm. Consequently, the tissues were double-stained with uranyl acetate and lead citrate. The numbers of autophagosome around the epithelial cells were observed via a TEM (JEM-1200EX, JEOL, Tokyo, Japan).

### 2.10. Statistical Analysis

Data were expressed as mean ± SD except specifically mentioned. Differences were considered significant when *P* < 0.05. Statistical analysis was performed using one-way ANOVA followed with statistical software (SPSS22.0, Chicago, IL, US) and presented by GraphPad Prism 6.0 (Diego, CA, US).

## 3. Results

### 3.1. Results of Screening the Targets of Friedelin and UC

Seventeen friedelin-related targets were obtained, 1111 UC-related targets were overlapped from Gene Cards database [25] and DrugBank database [26]. Then, we acquired 10 integrated targets of friedelin in the treatment of UC (Table 1) for further analysis.

### 3.2. Construction of PPI Network

**Figure 1:**
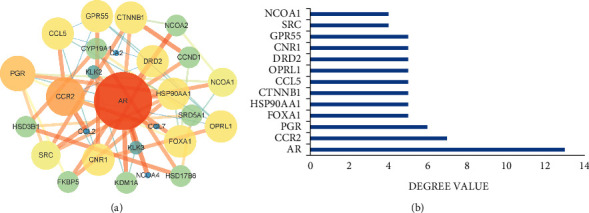
Construction of PPI network identification of core targets. (a) PPI network based on targets of friedelin in the treatment of ulcerative colitis. (b) Core targets in the PPI network.

The PPI network comprised 30 nodes and 53 edges (Figure 1(a)) was constructed to clarify the relationship between friedelin and UC. The degree of average node is 3.53. The size of the nodes in the interactive PPI network represented the degree of the nodes. The higher the degree is, the larger the size of the nodes is. The width of the lines in the interactive PPI network represented the connection between the genes. The closer the connection is, the wider the width of the lines is. 13 core targets (Figure 1(b)) were revealed based on twice the median degree. The 13 core targets were AR, CCR2, PGR, FOX1A, HSP90AA1, CTNNB1, CCL5, OPRL1, DRD2, CNR1, GPR55, SRC, and NCOA1. The degrees of the nodes were 13, 7, 6, 5, and 4.

### 3.3. Results of Molecular Docking

**Figure 2:**
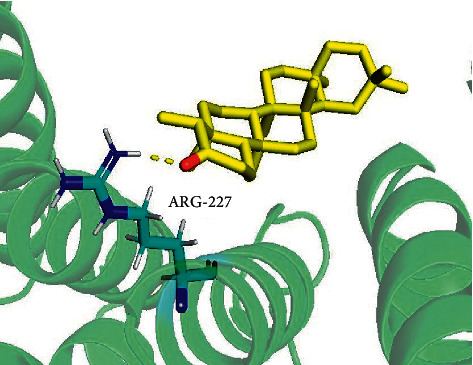
Docking locus and binding hydrogen bond of friedelin and AR.

Based on PPI network results, we chose the top 10 targets of 13 core targets: AR, CCR2, PGR, FOX1A, HSP90AA1, CTNNB1, CCL5, OPRL1, DRD2, and CNR1 for molecular docking. The results of docking (Table 2) showed that friedelin can integrate with the targets spontaneously. Indicated that friedelin plays a key role in the treatment of UC. Friedelin combined with proteins through hydrogen. For instance, friedelin combined with AR through ARG-227 (Figure 2).

### 3.4. Enrichment Analyses

**Figure 3:**
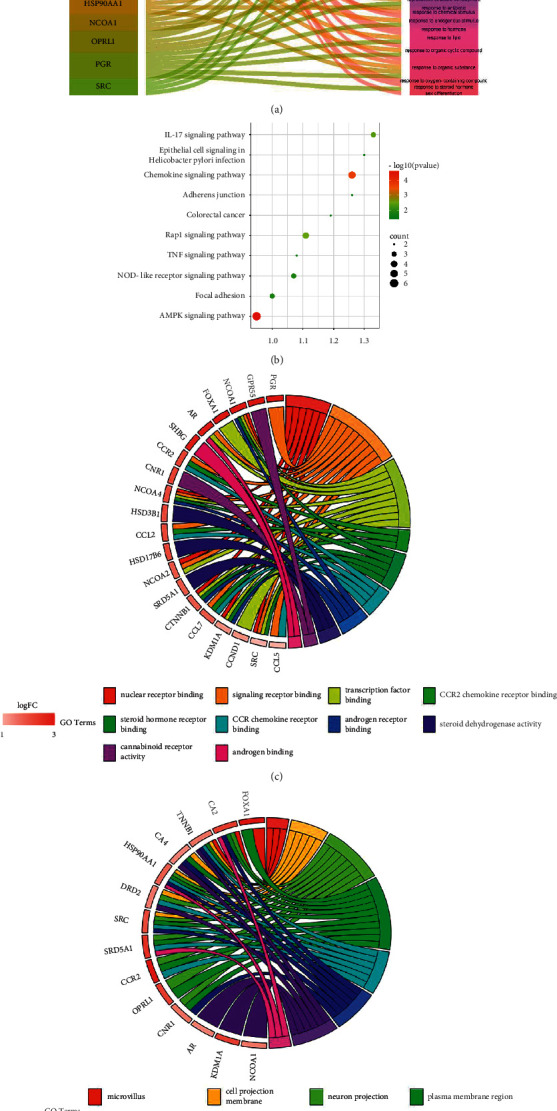
GO function and KEGG pathway analysis of network pharmacology. (a) Sankey diagram of the molecular function enriched for the targets. The order of molecular function from the top to the bottom is listed in supplementary material. (b) Signaling pathways of friedelin in the treatment of UC. (c, d) GO-BP and GO-CC of friedelin in the treatment of UC. (e) Signaling pathway in regard to AMPK pathway and autophagy, according to the enrichment analysis.

GO function and KEGG pathway enrichment analyses were performed to predict the mechanism of friedelin against UC. A total of 109 molecular functions (Figure 3(a)) were enriched based on *P* < 0.05; we noted friedelin attended in autophagy process in the treatment of UC. A total of 53 cellular components (Figure 3(b)) and 967 biological processes (Figure 3(c)) were enriched based on *P* < 0.05, containing cell periphery, response to oxygen-containing compound, and positive regulation of cell communication. 37 KEGG pathways were enriched containing AMPK signaling pathway, TNF signaling pathway, and IL-17 signaling pathway (Figure 3(d)). We concluded a signaling pathway in regard to AMPK pathway and autophagy, according to the enrichment analysis for further analysis (Figure 3(e)).

### 3.5. Friedelin Alleviated the Clinical Symptoms of Mice

**Figure 4:**
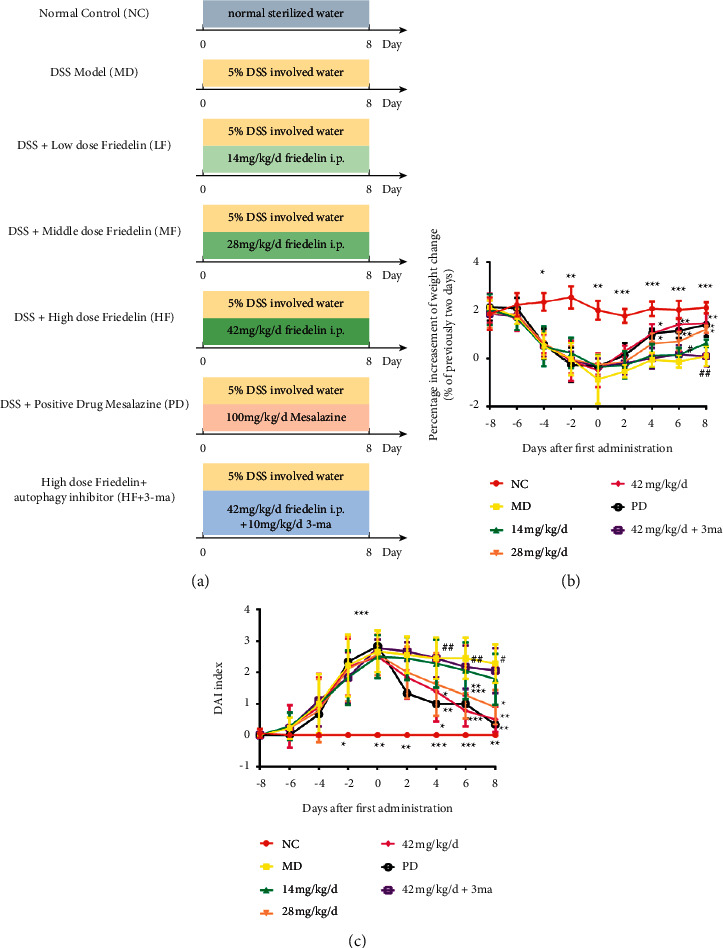
Friedelin promoted recovery in DSS-induced mice. (a) The protocol of this animal study. (b) Body weights during the animal study. (c) DAI scores. Statistical differences compared with the MD group were considered at ^*∗*^*P* < 0.05, ^*∗∗*^*P* < 0.01, or ^*∗∗∗*^*P* < 0.001. Statistical differences compared between the 42 mg/kg group and the 42 mg/kg^+3^ ma group were considered at #*P* < 0.05 or ##*P* < 0.01.

DSS was used to trigger UC model in mice, and friedelin was the intervention; mesalazine was the positive drug, and 3 ma was the common autophagy inhibitor (Figure 4(a)). Mice in the normal control group maintained a stable increasing weight during the experimental process, whereas mice in the DSS model group significantly suffered from weight loss due to malnutrition because DSS elicited an acute colitis (Figure 4(b)). Mice in the friedelin treatment groups attenuated the weight loss, indicating that the friedelin promoted the recovery of colon and the total situation of mice. Furthermore, DAI was an integrated index reflecting the overall severity of colitis; for example, 4 means most severe and 0 means no symptoms of hemoccult. Mice in the control group had low DAI score because the DAI score consists of symptoms of weight loss and bloody and loose stool. The DSS administration elicited these symptoms and therefore a rather high DAI score in the DSS model group. However, friedelin ameliorated these situations gradually (Figure 4(c)).

### 3.6. Friedelin Reserved Colon Length and Changed Pathology in Mice

**Figure 5:**
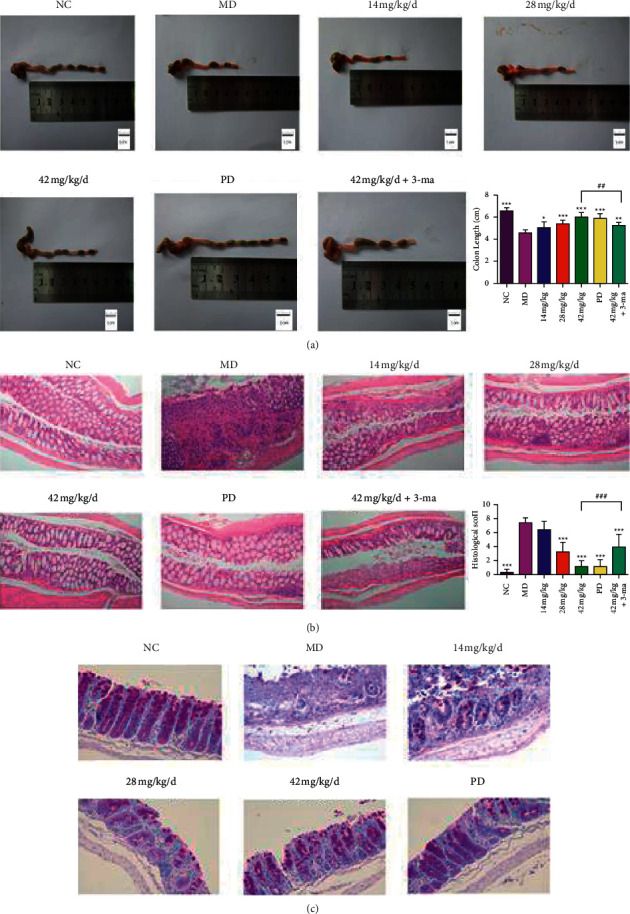
Friedelin promoted recovery in DSS-induced mice in histological behavior. (a) The colon length of all groups. (b) The HE staining of colon and HE histological scores of all groups. (c) The PAS staining of colon. Scale bar = 50 *μ*m. Statistical differences compared with the MD group were considered at ^*∗*^*P* < 0.05, ^*∗∗*^*P* < 0.01, or ^*∗∗∗*^*P* < 0.001. Statistical differences comparing between the 42 mg/kg group and the 42 mg/kg^+3^ ma group were considered at ##*P* < 0.01 or ###*P* < 0.001.

The colon length was firstly pictured and recorded. The colon length in control group is similar, whereas the colon length in the DSS model group suffered from a marked decrease in a dose-dependent manner (Figure 5(a)). The friedelin ameliorated the decrease of colon length in a dose-dependent manner. Furthermore, histological observation of HE staining (Figure 5(b)) demonstrated that friedelin ameliorated the colon inflammation. DSS elicited the injuries in colon, for example, the ulcer or inflammatory cell infiltration, erosion of epithelial layer, and loss of glandular epithelium compared the structure of colon in control group. The histological scores showed the friedelin groups had less ulcers and epithelial defects in a dose-dependent manner (Figure 5(b)). In addition, PAS staining demonstrated the severe decrease of goblet cells in DSS-induced mice, which was attenuated by friedelin administration (Figure 5(c)). These results exhibited the alleviation of friedelin on DSS-induced colitis in mice.

### 3.7. Friedelin Downregulated the Proinflammatory Cytokines in Mice Colon

**Figure 6:**
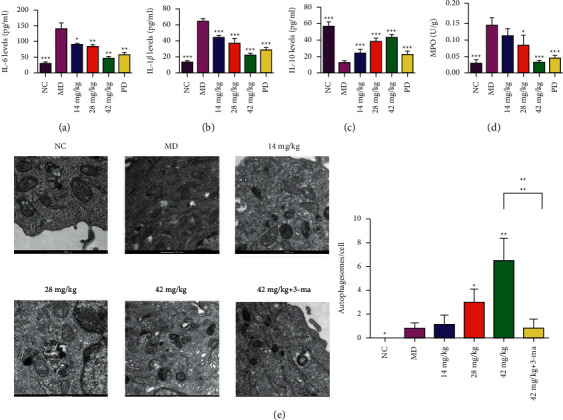
The proinflammatory and anti-inflammatory cytokines levels, MPO levels, and autophagosome observation of DSS-induced mice. (a) The IL-6 levels. (b) The IL-1*β* levels. (c) The IL-10 levels. (d) MPO values. (e) TEM observation of autophagosomes in all groups. Scale bar = 500 nm. Statistical differences compared with MD group were considered at ^*∗*^*P* < 0.05, ^*∗∗*^*P* < 0.01, or ^*∗∗∗*^*P* < 0.001. Statistical differences compared between the 42 mg/kg group and the 42 mg/kg^+3^ ma group were considered at ^*∗*^*P* < 0.05, ^*∗∗*^*P* < 0.01, or ^*∗∗∗*^*P* < 0.001.

In line with evaluation of pathology, the proinflammatory cytokine IL-6 levels were maintained at relative low levels in the control group. After DSS administration, the IL-6 levels were upregulated in DSS model group. However, the IL-6 levels were downregulated in three treatment groups in a dose-dependent manner after friedelin 8 days' injection compared with DSS group (Figure 6(a)). Consistently, the IL-1*β* levels in mice colon were also downregulated after friedelin administration in a dose-dependent manner (Figure 6(b)). In contrast, the anti-inflammation cytokines IL-10 levels were enhanced after friedelin administration in a dose-dependent manner (Figure 6(c)). Furthermore, the MPO activity was enhanced in the DSS group and downregulated after friedelin administration in a dose-dependent manner (Figure 6(d)). These results indicated that friedelin administration attenuated the DSS-induced inflammation in mice colon.

### 3.8. Friedelin Elicited the Autophagy in Mice Colon Epithelial Cells

**Figure 7:**
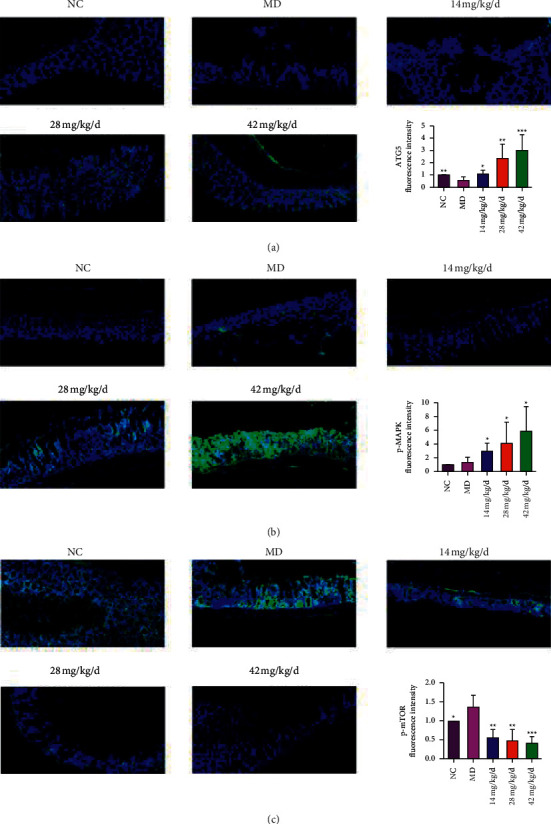
Friedelin regulated the DSS-induced autophagy-related proteins and AMPK-mTOR in DSS mice. (a) Friedelin activated levels of ATG5. (b) Friedelin activated levels of p-AMPK. (c) Friedelin inhibited levels of p-mTOR. Scale bar = 50 *μ*m. Statistical differences compared with MD group were considered at ^*∗*^*P* < 0.05, ^*∗∗*^*P* < 0.01, or ^*∗∗∗*^*P* < 0.001. Statistical differences compared between the 42 mg/kg group and the 42 mg/kg^+3^ ma group were considered at ^*∗*^*P* < 0.05, ^*∗∗*^*P* < 0.01, or ^*∗∗∗*^*P* < 0.001.

TEM result indicated that there were few autophagosomes in mice in the control group. After 8 days of administration, friedelin upregulated the numbers of autophagosome. These results suggested the direct evidence of friedelin could activate autophagy in mice colon epithelial cells (Figure 6(e)). IF results suggested partly intrinsic mechanism of friedelin administration. The ATG5 autophagy-related proteins were downregulated by DSS administration and further attenuated by friedelin administration (Figure 7(a)). Furthermore, the phosphorylated AMPK proteins activity levels were enhanced by friedelin administration (Figure 7(b)); the phosphorylated mTOR proteins activity levels were inhibited by friedelin administration (Figure 7(c)). Therefore, AMPK-mTOR signaling pathway serves as important roles in the friedelin's effect on DSS-induced UC model mice.

## 4. Discussion

The conventional therapy for UC is mainly based on the anti-inflammatory medicine. The anti-inflammatory drug research for UC was initiated in lab and further extended to clinical in the past decades. However, this strong efficacy is still lacked, and some side effect still exists in clinic. Indeed, there is a wide range of potential for traditional Chinese medicine in the treatment of UC. Therefore, this study investigated the potential mechanism and verified the exact molecular function of friedelin in the treatment of UC.

In this study, 17 potential targets of friedelin and 1111 related targets of UC were identified. Construction of PPI network reviewed 30 related targets between friedelin and UC. 13 core targets were further screened based on degree value. We identified top 10 targets from PPI network by molecular docking, further proving friedelin attended in the treatment of UC.

GO functional analysis revealed that the core targets were primarily related to autophagy, cell periphery, response to oxygen-containing compound, and positive regulation of cell communication. KEGG pathway analysis further revealed that the targets were primarily involved in inflammation and immunity pathways, such as the AMPK signaling pathway, TNF signaling pathway, and IL-17 signaling pathway. Moreover, amounts of research verified AR promoted autophagy in other diseases [40, 41]. Similarly, animal-based experiments demonstrated that friedelin could treat UC by inhibiting inflammation and improving autophagy.

In vivo, apparently high score of DAI in colitis mice suggested successful establishment of UC model in mice. The therapeutic activity of friedelin indicated that mice with treatment of friedelin had significant lower DAI index than that in model. Furthermore, the effect of friedelin on the length of colon was witnessed. Friedelin induced rise of autophagy levels via the upregulation of ATG5 levels. In addition, the AMPK/mTOR-dependent autophagy ameliorated the colon inflammatory response.

Autophagy is a regulated initiative cell degradation process that is responsible for the turnover of intracellular proteins and the damaged or incompetent organelles in cell. It could be induced by stress situations, such as hypoxia, starvation, and reticular stress. Autophagic dysfunction is recognized as the main contributing factor in a variety of chronic inflammatory diseases [42]. In 2007, a genome-wide association study of autophagy-related 16-like 1 (ATG16L1) showed the relationship between autophagy and IBD [43]. Since then, more autophagy-related protein activities were explored in UC. The Beclin1 protein activity in the colonic mucosa tissues of patients with UC was higher than normal people and tightly linked to the severity of UC [44]. Nowadays, the link of autophagy to UC is a topic that attracts much focus for new treatment strategy and new drugs promising and also some debate. Every coin has two sides. Some studies revealed that autophagy may contribute to the development of intestinal inflammation and some drugs or molecular target was developed to against autophagy, such as curcumin [45], Jian pi qing chang decoction [46], erbin [47], M10 [48], and miR-29a [49]. On the contrary, study showed that autophagy can prevent colitis by maintaining normal intestinal flora and mucus secretion [50]. Epithelial cell autophagy plays critical roles against colitis in mice [51]. Moreover, study revealed that reduced autophagy in epithelial cells can further develop the UC symptoms [52]. Recently study revealed that nicotine stimulation could ameliorate the colitis via enhancing autophagy [53].

Moreover, the friedelin markedly inhibited the production of excessive proinflammatory cytokines. The inflammatory response plays crucial roles in the UC pathogen. The TNF can elicit the intestinal mucosal impairment, while the IL-6 and IL-1**β** are important pathological mediators of UC [54, 55]. The MPO is a crucial index which implies the function of neutrophils because it is a lysosome maintained in the azurophilic granule of myeloid cells [56]. Inhibition of proinflammatory mediators can retard the severity of diarrhea, hematochezia, and colitis. Therefore, neutralization of these proinflammatory mediators was crucial molecular target of numerous drugs for UC treatment. As friedelin was reported with gastroprotective and anti-inflammatory activity mentioned before, it tempted us to administer friedelin in a DSS-induced UC mice model to verify the colon protective effect. In this study, friedelin downregulated the proinflammatory cytokines and MPO values. Meanwhile, IL-10 is an important anti-inflammatory mediator, which plays anticolitis roles in the UC pathogen [57]. The friedelin significantly upregulated the IL-10 levels in the colon tissues, which could exert a protective effect. Therefore, the upregulation of protective effect and downregulation of proinflammatory cytokines effect elicited by friedelin changed the immunological programming, which consequently improved colon environment in DSS-induced UC model mice. The comparison of 3 ma combination with friedelin with sole administration of friedelin validated the role of autophagy.

In our study, the in vivo inhibitor treatment using 3 ma combined the 42 mg/kg friedelin demonstrated role of autophagy in friedelin's pharmacological effect. Firstly, 3 ma inhibited the upregulation of autophagosome numbers by friedelin, which demonstrated the successful inhibition of autophagy. In addition, inhibition of autophagy using 3 ma attenuated the colon length in friedelin administrated UC mice. The DAI and pathology of mice colon were aggravated in 3 ma combination group. These results demonstrated that the autophagy serves an important role in protective ability of friedelin. In this study, friedelin induced upregulation of autophagy protein expression, then consequently inhibited inflammatory response, and promoted intestinal mucosal repair in UC.

The ATG12 conjugation system (ATG7, ATG10, ATG12, ATG16L1, and ATG5) was autophagy-related (ATG) proteins playing critical roles in the autophagy process. Among the above proteins, ATG5 is the center regulator for autophagic vesicle formation and can terminate or elicit the autophagosome in inflammation or neurological disease [58, 59]. Therefore, ATG5 can be employed to monitor the autophagy flux or autophagy levels. In this study, friedelin can elicit the ATG5 levels in colon epithelial cells; combined with TEM results, the induction effect of friedelin on autophagy could be validated. Previously, literature demonstrated that the AMPK was activated in cell energy supply abnormal or excess pressure, especially in low glucose, hypoxia, and toxicity [60]. AMPK signaling pathway is a critical signaling pathway in the metabolic process. Hence, AMPK signaling pathway commonly regulates the autophagy, a metabolic state of cell, which also participates in the intracellular energy metabolism. Activated AMPK can negatively modulate mTOR, which is a key modulator in the process of autophagy induction, consequently upregulated the autophagy levels in oceans of cells and diseases, such as tumor [61], hepatic steatosis [62], and COPD [63]. In this study, friedelin could elicit the AMPK phosphorylation activation and the negatively inhibition of mTOR phosphorylation. Therefore, AMPK-mTOR signaling pathway acted as a key role in the pharmacologic effect of friedelin.

There are some innovations and limitations in this study. The main innovations are as follows. (1) This study investigates the effect of friedelin on autophagy to regulate UC. This is different from many previous drugs, which are only focused on as anti-inflammatory. (2) Different from the traditional speculation or preexperiment to detect possible mechanisms and targets, we predicted that the drug might regulate autophagy targets through AMPK through network pharmacological research and verified the prediction in the experiment. The main limitations are as follows. (1) According to the prediction of network pharmacology, TNF signaling pathway and IL-17 signaling pathway may also be in the mechanism of action of friedelin, which may be related to its anti-inflammatory properties. However, this study is limited and does not make in-depth exploration. (2) In recent years, there are other mechanisms for the regulation of UC, such as microbiota regulation, but this study has not explored it.

## 5. Conclusion

The potential targets and mechanism of friedelin in treating UC were successfully identified by network pharmacology and molecular docking. Moreover, the present study revealed that friedelin, a natural bioactive agent, ameliorated the DSS-induced colitis in mice model. Friedelin downregulated the inflammatory cytokines levels, promoted the recovery of colon mucosa, and consequently enhanced the improvement of symptoms and maintenance of body weight. Moreover, autophagy promotion played important roles in the friedelin's mechanism. Our study suggested that friedelin could be employed as a potential candidate for UC treatment.

## Figures and Tables

**Table 1:** The integrated targets between friedelin and UC.

Target	Common name	UniProt ID	Degree value
Androgen receptor	AR	P10275	13
Cyclooxygenase-1	PTGS1	P23219	12
Steroid 5-alpha-reductase 1	SRD5A1	P18405	10
Carbonic anhydrase II	CA2	P00918	7
C–C chemokine receptor type 2	CCR2	P41597	7
Cannabinoid receptor 1	CNR1	P21554	7
Testis-specific androgen-binding protein	SHBG	P04278	7
Progesterone receptor	PGR	P06401	6
Cytochrome P450 19A1	CYP19A1	P11511	5
Carbonic anhydrase IV	CA4	P22748	5

**Table 2:** The affinity scores and docking sites of friedelin with the top 10 targets.

	Affinity	*X*	*Y*	*Z*	Size *x*	Size *y*	Size *z*
AR 3BTR	−8.34	32.541	81.756	61.276	13.218	12.890	12.753
CCR2 5UIW	−6.85	150.718	103.976	630.414	11.378	11.240	24.400
PGR 2C7A	−7.84	10.363	52.041	51.231	31.591	27.780	55.927
FOXA1 5A5U	−8.933	338.583	365.265	224.676	45.232	25.417	74.761
CYP19A1 5JKV	−6.07	96.632	38.415	35.468	14.995	18.693	14.886
CTNNB1 2G57	−7.03	6.767	2.323	−1.656	17.444	13.376	15.054
CCL5 5L2U	−4.17	10.312	217.485	32.492	14.998	16.365	11.566
OPRL1 5DHG	−9.36	−8.950	27.186	4.709	19.255	25.242	17.370
DRD2 6CM4	−10.33	9.186	4.863	−11.033	16.364	20.759	25.106
CNR1 5TGZ	−1.85	43.638	27.470	318.531	13.825	20.368	15.234

The PDB ID number of target protein was followed after target names. Affinity means the docking energy; the lower the affinity is, the tighter the combination between target protein and friedelin is. *X*, *Y*, and *Z* mean the docking sites, and size *x*, size *y*, and size *z* mean the docking range based on docking sites.

## Data Availability

The data of this study can be obtained from the corresponding author upon rational request.

## Supplementary Materials

Supplementary Materials Figure S1: graphical abstract of the article.
