# Supplementary figures and images for: Revealing the Mechanism of Friedelin in the Treatment of Ulcerative Colitis Based on Network Pharmacology and Experimental Verification
Source: Evid Based Complement Alternat Med. 2021 Nov 2;2021:4451779. doi: 10.1155/2021/4451779 (PMC8577922; doi:10.1155/2021/4451779)

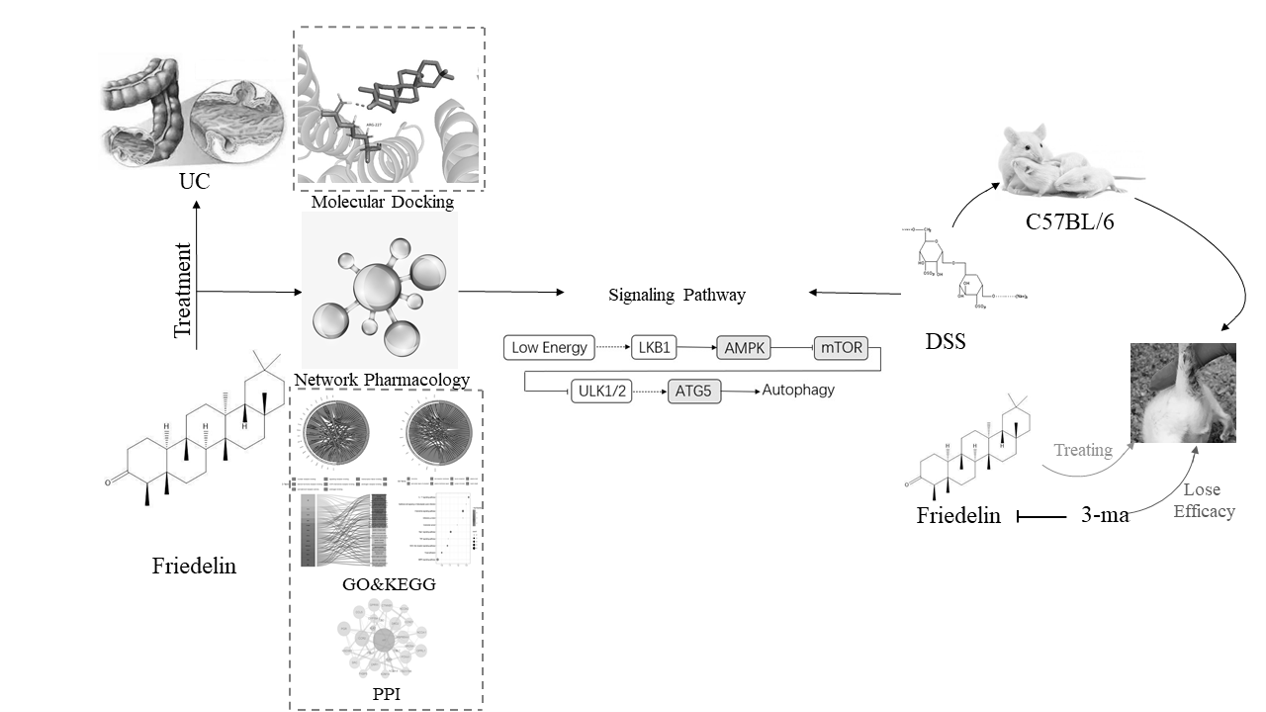

Supplement: Supplementary Materials — Figure S1: graphical abstract of the article. [file 4451779.f1.png]
